# An update on immunological and molecular tests and their impact in infectious uveitis

**DOI:** 10.3389/fopht.2023.1132131

**Published:** 2023-03-21

**Authors:** Joanne Thomas, Nam V. Nguyen, Tolulope Fashina, Ye Huang, Steven Yeh, Christopher D. Conrady

**Affiliations:** ^1^ Medical College of Georgia, Augusta University, Augusta, GA, United States; ^2^ College of Medicine, University of Nebraska Medical Center, Omaha, NE, United States; ^3^ Department of Ophthalmology and Visual Sciences, Stanley M. Truhlsen Eye Institute, University of Nebraska Medical Center, Omaha, NE, United States; ^4^ Department of Pathology and Microbiology, University of Nebraska Medical Center, Omaha, NE, United States

**Keywords:** polymerase chain reaction (PCR), immunological testing, molecular diagnosis, next-generation sequencing, uveitis

## Abstract

Early diagnosis of infectious uveitis can lead to prompt initiation of treatment to minimize vision-threatening sequelae. As various infectious etiologies of uveitis share similar clinical features, advancements in polymerase chain reaction (PCR) and metagenomic next-generation sequencing (MDS) have shown significant promise in improving diagnostic capabilities. Various techniques of PCR, including real-time, multiplex, comprehensive, and broad-range, have increased the armamentarium for infectious uveitis diagnosis. Additionally, metagenomic deep sequencing technology has provided a methodology to identify causative pathogens as well as novel etiologies of uveitis. This review discusses the diagnostic tools available for infectious uveitis and highlights the advantages and disadvantages of the techniques.

## Introduction

Uveitis is a sight-threatening inflammatory disorder of the eye that is broadly categorized into non-infectious or infectious etiologies. It is one of the leading causes of blindness, accounting for 15% of cases of total blindness in Western countries, with an annual incidence of 30,000 cases (1, 2). While non-infectious causes are more common in developed countries, infectious uveitis still accounts for 20% and 50% of all uveitis cases in the United States and developing countries, respectively (3, 4). A prompt diagnosis through clinical observation and directed serological and ocular fluid testing is needed to identify the underlying etiology. Unfortunately, in only approximately 50% of cases of presumed infectious uveitis can a pathogen be detected with standard culture methods highlighting the need for more rapid and sensitive diagnostic tools to improve the identification of causative organisms and guide treatment (5).

Since both infectious and non-infectious uveitis can cause a similar clinical presentation, an accurate determination of the etiology leads to prompt treatment and a reduction of long-term complications. Advances in techniques, including polymerase chain reaction (PCR) and metagenomic next-generation sequencing (MDS), aid in the diagnosis process and may lead to the identification of previously unknown causative organisms of infectious uveitis (6–9). This review provides an overview of current diagnostic modalities for infectious uveitis and diagnostic techniques on the horizon that could improve diagnostic yields in a uveitis evaluation.

## Infectious Entities of Uveitis

While various bacterial and protozoal organisms can manifest with uveitis, uveitis due to tuberculosis, syphilis, and toxoplasmosis are commonly reported. Ocular involvement of *Mycobacterium tuberculosis* most commonly presents as posterior uveitis (TPU) but can present as granulomatous anterior uveitis, intermediate, and panuveitis (10). *Treponema pallidum* associated uveitis manifests as posterior uveitis or non-granulomatous anterior uveitis although variable presentations are possible (11). Additionally, uveitis may be the initial manifestation of syphilis (12). Ocular toxoplasmosis due to *Toxoplasma gondii* is the leading cause of posterior uveitis globally (13). This presents as necrotizing retinitis mostly in the macula with an overlying vitreous haze causing a “headlight in the fog” appearance.

The most common viruses causing uveitis are from the Herpesviridae family: herpes simplex virus (HSV) type -1 and -2, cytomegalovirus (CMV), and varicella zoster virus (VZV) (14, 15). These infections can cause sight-threatening ocular complications, including keratitis, hypertensive anterior uveitis, acute retinal necrosis (ARN), and progressive outer retinal necrosis (PORN) (15). Although the leading cause of ARN is VZV, other viruses have been reported (16). ARN is characterized by granulomatous panuveitis, retinal arteritis, and necrotizing retinitis, which has areas of yellowish necrotic lesions starting peripherally and expanding centripetally (15). While ARN is largely a clinical diagnosis with diagnostic criteria set by the American Uveitis Society (17), PCR and other diagnostic modalities can confirm the specific pathogen. PORN is a variant of ARN usually seen in immunocompromised patients and can differ from that of ARN as it may start posteriorly rather than peripherally (15). Like ARN, PORN is a clinical diagnosis, but testing for viral etiologies through serology or PCR is important in identifying the causative pathogen.

CMV retinitis occurs mostly in immunocompromised patients and is typically characterized by full-thickness, perivascular yellow-white necrotizing retinitis, associated hemorrhages, periphlebitis, and minimal overlying vitritis (15). Although diagnosis is usually clinical, PCR analysis for CMV retinitis can assist in managing and monitoring response to treatment. While differences in clinical presentations of herpes infections of the posterior segment may be appreciated, findings in anterior uveitis from these entities are more discrete, and additional diagnostic testing of ocular fluid is required for diagnosis and treatment. There can also be significant overlap in the clinical presentation of bacterial and fungal infections of the eye, and while less established than with viral etiologies, PCR testing to identify these pathogens is becoming more commonplace. **Table 1** summarizes infectious causes of uveitis discussed herein.

**Table 1:** Common Infectious Causes of Uveitis.

Infectious Entity	Type of Organism	Characteristics of Uveitis
Mycobacterium tuberculosis (10)	Bacterial	-posterior uveitis (most common) -granulomatous anterior uveitis -intermediate uveitis
Treponema pallidum (11)	Bacterial	-posterior uveitis (most common) -non-granulomatous anterior uveitis -variable presentations possible
Toxoplasma gondii (13)	Protozoal	-posterior uveitis (leading cause globally) -necrotizing retinitis with overlying haze (“headlight in the fog”)
Herpes simplex virus (HSV-1 and 2) (18)	Viral	-ARN can be due to HSV (most common after VZV) -HSV-2 associated ARN is more common in children
Cytomegalovirus (CMV) (15)	Viral	-more common in immunocompromised patients -full-thickness, perivascular yellow-white necrotizing retinitis -PORN is seen more in immunocompromised patients
Varicella Zoster Virus (VZV) (16)	Viral	-leading cause of ARN
SARS-CoV-2 (19)	Viral	-anterior uveitis more common -case of MEWDS reported

## Polymerase chain reaction principles

The practice of diagnosing uveitis has changed with the development and widespread use of PCR. The technique was developed in the 1980s and involves rapid amplification of target DNA sequences by utilizing DNA polymerases and cycles of temperature changes (20). The technique has expanded to target RNA sequences [reverse transcription (RT)-PCR] and can be quantitative or qualitative, depending on assay design. Multiple PCR strategies are available, included nested, multiplex, or real-time, which may be utilized in specific clinical circumstances. With all PCR techniques, contamination is possible, or even the detection of low levels of pathogenic DNA that may be within “normal limits,” either of which could lead to false positive results (21). Thus, there is some concern that there may be issues differentiating “contaminants” versus causative pathogens.

## Types of PCR testing

### Real-time PCR and multiplex PCR

Real-time PCR can be quantitative or qualitative and is used to determine the amount of a specific nucleic acid sequence in a biological sample, which may correlate to disease severity (22, 23). Multiplex PCR, a technique utilizing numerous primer pairs within a single reaction, is used in qualitative analysis to quickly screen a panel of different viruses, bacteria, and fungi. However, since this is a qualitative technique, it is unable to measure copy number to determine pathogen burden and cannot be used to monitor for therapeutic response over time (20). In a study by *Kumar et al.* sensitivity of multiplex PCR was 85.2% and specificity was 97.8%. Positive predictive value (PPV) was 87.9% and negative predictive value (NPV) was 97.2% (24).

### Comprehensive PCR

A combination of multiplex PCR and real-time PCR, named comprehensive PCR, has been developed to diagnose uveitis (20). With this approach, the multiplex assay initially screens for the presence of DNA from various pathogens. The identified pathogen sequences are then amplified by real-time PCR to quantify the amount of nucleic acid within the sample. A prospective case series from *Sugita et al.* evaluated the diagnostic parameters of comprehensive PCR and found sensitivity at 91.3%, specificity at 98.8%, PPV at 98.5%, NPV at 92.4% (25).

Recently, the multiplex solid-phase PCR strip kit was developed as a new comprehensive PCR assay for infectious uveitis. This strip PCR can target several pathogens, including herpesvirus, human T-lymphotropic virus (HTLV-1), *Mycobacterium tuberculosis*, *Toxoplasma gondii*, and *Candida albicans (* 20 *).* The main advantage of this assay is the detection of 24 different pathogens in one assay allowing for more rapid diagnosis, reproducibility, and ease of use (20). A study by *Nakano et al.* optimized the original 24-pathogen strip PCR assay for nine pathogens. These nine pathogens were HSV-1, HSV-2, VZV, HTLV-1, Human herpes virus (HHV)-6, Epstein-Barr virus (EBV), CMV, *Toxoplasma gondii*, and *Treponema pallidum* (26). The optimized strip PCR detected DNA at concentrations from 10 (0) to 10 (9) copies/mL in 252 out of 255 positive samples, as noted by conventional single-target PCR (26). This strip PCR was shown to have low intra- and inter-institutional variability with both beginner and expert users (26). Sensitivity was 98.8%, and specificity was 98.5%. PPV was 98.8% with NPV at 98.5% (26).

### Broad-range PCR

To assess for the presence of bacterial or fungal DNA, broad-range PCR can be utilized. This technique uses primers targeting the highly conserved 16s ribosomal DNA (rDNA) in bacteria and 18S or 28S rDNA in fungi (20). This allows for identification of the potential pathogen by comparing the amplified rDNA to nucleotide databases. Subsequent real-time PCR can quantify the target DNA sequences (20, 27–30).

Broad-range, real-time PCR correctly detected the underlying fungal etiology in 10 of 11 cases of confirmed fungal endophthalmitis (31). Detection of the bacterial conserved region using broad-range real-time PCR showed that it was positive in 95% (18/19) of patients diagnosed with bacterial endophthalmitis but positive in only 6% (3/50) of the non-infectious uveitis control patients (32). False negative results for bacterial endophthalmitis using this PCR technique occurred mostly in aqueous humor samples (20). False positive results were seen in some cases of idiopathic uveitis, but the copy number was lower showing the importance of utilizing copy number information for interpretation. To mitigate the risk of false positive results due to contamination, there have been suggestions of setting a cut-off value for the broad-range, real-time PCR assays to be considered positive at >100 copies/mL (20, 31, 32). If concerns about contamination and false positive results are present, other diagnostic modalities, such as cultures and serum antibody titers, should be utilized for confirmation.

## Studies on the role of PCR in diagnosis of infectious uveitis

In recent years, PCR has been instrumental in diagnosing several pathogens previously unknown to cause ocular disease, including *Trypanosoma cruzi* and HTLV, and differentiating members of virus families (6, 33). HHVs are associated with anterior and/or posterior uveitis. They can be detected and differentiated by PCR of ocular fluid utilizing HHV-specific primers to detect genomic DNA of HSV-1 or -2 (HHV 1-2) (28, 29), VZV (HHV 3) (28–30), and CMV (HHV 5) (27, 29, 34). PCR is important in differentiating these pathogens as they share many clinical features (20, 29). Prior to the widespread use of PCR, a diagnosis of ARN was based on clinical presentation, exam findings, and the Goldmann-Witmer coefficient (GWC), which compares intraocular antibody titers to that of serum (35). PCR is faster and more sensitive than GWC, especially in immunosuppressed patients with poor antibody production and is now more commonly utilized for ARN diagnosis (36, 37). In some cases, PCR may be used with GWC to improve diagnostic yield (36, 37).

Besides HSV-1 and -2, CMV and VZV, PCR has detected EBV, another herpesvirus, in 17 out of 500 (3.4%) patients with infectious uveitis in a prospective clinical case series (25). As PCR has increased in use, high concentrations of HHV6 DNA have been detected in ocular fluid leading to identification of two variants of HHV6, type A and B, suggesting that viral replication of this virus may occur even in the eye (20, 38). There is limited data on HHV7 or HHV8 and the eye, but there are emerging case reports of both pathogens causing endotheliitis (38, 39). Additionally, PCR has led to advancements in the identification of ocular tuberculosis by using conserved sequences such as IS*6110* or the predominant *mpb64* gene in mycobacterial genome (40) as well as has assisted in the diagnosis of ocular toxoplasmosis.

HTLV-1 can cause uveitis and is especially common in certain areas of Japan (20). Studies have shown detection of the virus in intraocular fluid by PCR supporting HTLV-1-associated uveitis as a distinct clinical entity (41). The diagnosis of HTLV-1-associated uveitis is based on the exclusion of other causes of uveitis, PCR positivity for the virus, and the presence of antibodies to the pathogen (20). SARS-CoV-2 associated uveitis (42, 43) has been reported including a case of Multiple Evanescent White Dot Syndrome (MEWDS) (19), and PCR has been utilized especially to test tear fluid for SARS-CoV-2 (44, 45). In addition, other viral diseases such as Ebola, Dengue (46), West Nile (47), Zika (48), and Chikungunya (49) are better appreciated as potential ocular pathogens due to the use of PCR to identify viruses historically not thought to cause eye disease.

PCR can also be used for detection of ocular toxoplasmosis in ocular fluid (20). A study conducted by *Sugita et al.* showed that a two-step PCR using qualitative multiplex PCR and real-time PCR detected *T. gondii* in all patients with active uveitis from the pathogen (50). GWC can also be useful for diagnosing ocular toxoplasmosis but is no longer routinely used due to the sensitivity of PCR (14). However, with a combined approach utilizing GWC and PCR, diagnostic sensitivity improves from 38% to 93% (51).

## Advantages and disadvantages of PCR

Given the need for prompt treatment in infectious uveitis due to host tissue damage, PCR can provide results more rapidly than eye cultures, as the sample-to-answer time can occur within hours. Traditional PCR has been shown to have similar detection rates for infectious uveitis as routine cultures (38.2% for cultures versus 34.6% for PCR in aqueous humor samples; 54% versus 57% in vitreous samples) but has a substantially better detection rate than standard microbiological techniques in cases where intravitreal antimicrobials have been previously administered (PCR 70% vs. 9% respectively) (52).

Obstacles to widespread PCR use exist, given the need for laboratory resources and personnel to perform the technique, including availability of required reagents and materials. Serial testing for multiple pathogens can become expensive and time-consuming if multiplex PCR is unavailable. Additionally, PCR testing should ideally be directed by a patient’s clinical presentation and risk factors (e.g., perivascular retinal whitening with hemorrhages and history of human immunodeficiency virus makes CMV retinitis likely), as specific primers are needed to run the test targeting the pathogen. Because PCR amplification may detect contaminants more readily, quantitative analysis and interpretation of cycling thresholds may be necessary if qualitative techniques are initially used.

## Emerging diagnostics: Metagenomic next generation deep sequencing

Since its introduction in the early 2000s, next generation sequencing has reduced sequencing time of large DNA fragments and/or genomes. MDS allows rapid sequencing of multiple sequences simultaneously, thus reducing processing time of a specimen compared to more traditional methods. With this approach, the clinical specimen undergoes nucleic acid sequencing, and the resulting sequences are mapped and compared to a library of reference genomes for pathogen identification (53). Therefore, this technique can be used to map nucleic acid sequences and identify the pathogen causing uveitis.

Although MDS can screen a wide range of infectious etiologies, pathogen-directed PCR is utilized more commonly for diagnostic purposes due to its cost-effectiveness and superior time efficiency. Numerous studies have been performed at the University of California San Francisco (UCSF) Proctor Foundation looking at DNA and RNA MDS for diagnosis of intraocular infections (5, 8). Their protocol for RNA analysis involves RNA extraction from a sample of intraocular fluid (aqueous or vitreous) and synthesis of double-stranded complementary DNA (cDNA). The cDNA is converted to libraries, amplified, and sequenced using 135 nucleotide paired-end sequencing, an advancement in improving efficiency and accuracy. Sequencing reads are mapped to the references and analyzed using a rapid computational pipeline developed by the DeRisi Laboratory (54). The pathogen profiles are identified by comparing results to the National Center for Biotechnology Information (NCBI) Sequence Read Archive (8). Similar to the MDS RNA sequencing process, MDS DNA sequencing involves amplification and sequence mapping and comparing it to nucleotide reference database (5).

## Studies on the role of metagenomic next generation sequencing in diagnosis of infectious uveitis

Due to its unbiased testing and broad diagnostic range, MDS can help to identify uncommon infectious pathogens causing uveitis. In contrast, traditional PCR techniques may not unless there is a high clinical suspicion for the specific organism. Uncommon pathogens such as Zika, Ebola, and Rubella viruses and *Leptospira santarosai* have been shown to cause uveitis; however, they are often not included in the standard uveitis PCR work-up panel (55–58). With its broad diagnostic range, MDS can help identify these uncommon pathogens to reduce ocular complications from delays in appropriate treatment.

A proof-of-concept study was done at the UCSF Proctor Foundation using MDS RNA sequencing to identify infectious etiologies of uveitis (8). Three subjects with known infectious etiologies, two subjects with non-infectious etiologies, and one subject with bilateral chronic uveitis without a known etiology were included in the study (8). Subjects with known etiologies were assessed by conventional PCR testing. Intraocular samples from all subjects were subjected to metagenomic deep sequencing. As identified *via* PCR, subjects positive for *Cryptococcus neoformans*, *Toxoplasma gondii*, and HSV-1 were correctly identified by MDS RNA sequencing. These patients’ clinical symptoms improved significantly upon treatments directed at the causative agents. Additionally, MDS RNA sequencing was able to identify Rubella virus (RV) as the causative agent in a subject with bilateral chronic uveitis. This finding was later corroborated with RT-PCR and positive serology (8). Although this study had a small sample size, it showed that MDS RNA sequencing is useful in diagnosing infectious uveitis. A similar study with a larger sample size was conducted on 41 intraocular samples from patients with presumed ocular infections (59). These samples went through the MDS RNA sequencing protocol and pathogen-directed PCRs in a masked manner. The positive percent agreement between RNA-seq and pathogen-directed PCRs was 100% (95% confidence interval (CI): 75.5% to 100%) (59). MDS RNA sequencing identified the causative agent in four intraocular samples that were missed by pathogen-directed PCRs, including a rare *Pithomyces* species. In another study, MDS RNA sequencing was able to identify RV in six patients with Fuchs heterochromic iridocyclitis (55).

The utility of MDS DNA sequencing in infectious uveitis was evaluated in a study of intraocular fluid of 31 positive-pathogen samples with PCR findings and 36 negative-pathogen samples (5). Out of 31 positive samples, 27 were identified correctly with MDS DNA sequencing, while four were negative. Out of 36 negative samples, DNA sequencing identified six different pathogens, which were not detected or tested by PCR, including CMV, HHV-6, HSV-2, HTLV-1, *Klebsiella pneumoniae*, and *Candida dubliniensis* (5). A case report by the same group identified *Leptospira santarosai* using MDS DNA sequencing. Targeted treatment was initiated and the uveitis resolved (56). Thus, there is emerging evidence that both RNA- and DNA-based MDS may provide important diagnostic information.

## Advantages and disadvantages of metagenomic next generation sequencing

MDS RNA and DNA sequencing are unbiased tests, and the results require interpretation with the clinical phenotype, while with traditional PCR testing, the molecular tests are based on clinical findings and pretest probability. While RNA samples present certain challenges for sample storage due to the temperature-sensitivity of RNA, DNA is more tolerant of temperature fluctuations; therefore, it can be more easily used for metagenomic sequencing (5). Although most institutions have access to sequencing platforms, reagent and sequencing costs for MDS are more costly and time-intensive than traditional PCR tests (53). The analysis is also labor-intensive, requiring bioinformatics expertise. With advances in technology, the process can be potentially automated to reduce cost and labor (60). Moreover, rare infectious causes of uveitis can be identified, and targeted treatments can be used to effectively prevent vision-threatening sequelae in patients with uveitis.

## Discussion

PCR and genome sequencing are revolutionizing modern medicine. While there are some drawbacks to PCR technologies and its derivatives, molecular diagnostics are particularly helpful in cases of pathogens, which are difficult to culture, smaller clinical volumes, and given the potential for an unexpected identification of previously unknown pathogens in a tissue. This is important in ophthalmology due to the increased sensitivity of PCR with smaller clinical samples and easier identification of novel pathogens compared to older techniques. While traditional PCR and RT-PCR are well-established for the confirmation of specimens with characteristic clinical presentations, the use of unbiased molecular techniques will likely play a greater role in the diagnostic armamentarium for infectious pathogens.

## Author contributions

NN and JT: These authors share first authorship. Conceptualization: SY. Investigation: NN, JT. Writing—original draft preparation: NN, JT. Writing—review and editing: CC, YH, TF, NN, JT, SY. Supervision: CC, SY. Funding acquisition: SY. All authors have read and agreed to the published version of the manuscript. All authors contributed to the article and approved the submitted version. 
